# The effect of system-level intervention in early childhood education and care on children’s dietary intake and environmental impact of the diet

**DOI:** 10.1007/s00394-026-04064-x

**Published:** 2026-07-17

**Authors:** Mari Åkerlund, Venla Kyttä, Jelena Meinilä, Arto Pietilä, Tuuli E. Korhonen, Henna Vepsäläinen, Liisa Korkalo, Satu Kinnunen, Sari Niinistö, Susanna Raulio, Leena Forma, Maijaliisa Erkkola, Merja Saarinen, Suvi M. Virtanen

**Affiliations:** 1 Department of Public Health, Finnish Institute for Health and Welfare, P.O. Box 30, FI-00271 Helsinki, Finland; 2 Unit of Health Sciences, Faculty of Social Sciences, Tampere University, Tampere, Finland; 3 Natural Resources Institute Finland, Latokartanonkaari 9, FI- 00790 Helsinki, Finland; 4 Department of Food and Nutrition, University of Helsinki, Helsinki, Finland; 5 Department of Health and Social Management, University of Eastern Finland, Kuopio, Finland; 6 Tampere University Hospital, Wellbeing Services County of Pirkanmaa, Tampere, Finland; 7 Center for Child, Adolescent and Maternal Health Research, Tampere University, Tampere, Finland

**Keywords:** Preschool, Daycare, Food consumption, Nutrient intake, Sustainability, Food education

## Abstract

**Purpose:**

Food systems have a large impact on human health and environmental sustainability. Early childhood education and care is an ideal setting for systemic change. We performed a cluster-randomised trial aiming at climate-friendly and healthy sustainable diet at 23 daycare centres in four Finnish municipalities in 2022 and evaluated system-level intervention effects on food consumption and nutrient intake, and climate and biodiversity impacts of the diet.

**Methods:**

The intervention was initially designed by the research group and refined through co-creation workshops with food service and daycare professionals, and municipal decision makers. The 10-month intervention comprised a modified menu towards more plant-based food supply, and food education. Children’s (*n* = 111) diet was assessed at baseline, mid-term, and/or at the end of the intervention by food frequency questionnaires. The climate and biodiversity impacts per megajoule were assessed using previously specified coefficients for the foods. Multilevel linear mixed models were used in the analysis.

**Results:**

During the intervention, children’s consumption of legumes and legume products and fish from nearby areas increased, while that of red meat, sausages, and other meat products decreased (Bonferroni-corrected *p* < 0.001). The changes in food consumption were substantial, with effect sizes ranging from 0.9 to 2.69. Nutrient intake was not affected by the intervention. Intervention decreased climate and biodiversity impacts of food provided at daycare (Bonferroni-corrected *p* < 0.001, *p* = 0.003, respectively).

**Conclusion:**

This study demonstrated that well-planned systemic change in meal provision and food education at daycare resulted in healthier and more climate-friendly diets among children.

**Supplementary Information:**

The online version contains supplementary material available at 10.1007/s00394-026-04064-x.

## Introduction

Food systems have a large impact on human health and environmental sustainability [1]. There is a growing international consensus supporting the transition toward a global standard - a more plant-based diet and more diverse use of protein sources in order to achieve the 2030 sustainable development goals, and in preventing non-communicable disease burden [1, 2]. In Finland, the environmental impacts of diet could also be significantly reduced by switching to a more plant-based diet, theoretically by up to 30–40% in terms of climate impact and 70% in terms of terrestrial biodiversity impact [3, 4]. Recently updated Nordic Nutrition Recommendations include environmental aspects due to the growing awareness of the environmental impacts of food [5]. Higher intakes of vegetables, legumes, fruits, berries, whole-grain cereals, and sustainable fish, and lower intakes of red and processed meat are encouraged by several expert bodies [5, 6].

All population groups can be recognised as actors for sustainable change. Particularly, young children are critical as childhood food habits may track to adulthood and may have long-term effects on health. As with adults, the current diet of the Finnish children is not conducive to environmental sustainability nor aligned with dietary guidelines [7, 8]. The typically high use of milk products, meat as a central component of meals, and a low consumption of vegetables, legumes, and sustainable fish are the key traits of the Finnish food culture that affect the food consumption of the children and would need to be challenged. The transformation towards a more climate-friendly and healthy diet would require major changes also in the food system [3, 9].

In Finland, early childhood education and care (ECEC) is an ideal setting for systemic change, since nearly all 3-5-year-olds (89% in 2022) [10] participate in it enabling daily meals served by food services and comprehensive food education. Children in ECEC centres are provided with meals based on the length of their attendance, for example, breakfast, lunch, and afternoon snack in full-time daycare. The food served in the ECEC should meet nutritional requirements (Act on Early Childhood Education and Care 540/2018, Chap. 2, 11 §) [11]. The meals are prepared by the food services located in the ECEC centres’ premises or delivered daily from the central kitchen. Food education, which consists of both planned, lesson-like, formal activities and more informal games, play, and discussions, is a part of daily pedagogic activities and holistic learning about wellbeing [12].

Long-term intervention designs at the system level are rare and would provide much-needed evidence on the safety and acceptability of more plant-based food for children. This study aimed to evaluate the effects of systemic dietary intervention at ECEC centres on food consumption and nutrient intake and on climate and biodiversity impacts of child’s diet eaten both at ECEC and home.

## Subjects and methods

One municipality from Päijät-Häme (Southern Finland) and three municipalities from South Ostrobothnia (Western Finland) agreed to participate in the FoodStep project in 2021. In each of the four participating municipalities, the food services of ECEC centres were operated by a single food service provider, all of which agreed to participate in the study. In one municipality, this provider was a private company, while in the other three municipalities, the food services were managed as part of the municipal organisation. Despite being a private company, the provider operates under municipal guidelines and delivers publicly commissioned services.

The ECEC centres (*n* = 23) were recruited in the study in autumn 2021. The number of children aged 3–5 years attending full-time care was considered. Fifteen ECEC centres from Päijät-Häme, and eight from South Ostrobothnia participated in the study. The ECEC centres from Päijät-Häme were cluster-randomised to intervention (*n* = 8) and control (*n* = 7) ECEC centres in February 2022. The randomisation (simple random allocation) into intervention and control ECEC centres was performed within four strata defined by the socio-economic profile of the areas. Among ECEC centres in South Ostrobothnia, randomisation was not feasible for practical reasons related to meal logistics and limited kitchen capacity, and intervention ECEC centres (*n* = 3) were instead selected based on the presence of an in-house kitchen. Of the children 34 came from South Ostrobothnia (31% of all 111). The control ECEC centres (*n* = 5) did not have an in-house kitchen, and food was transported to them.

### Intervention

The intervention contents were planned by the research group and further refined and adjusted during three co-creation workshops with the key stakeholders: food service and ECEC professionals and municipal decision makers.

The intervention consisted of modified menu, which was served to all children in the intervention ECEC centres, and food education. The implementation of the menu changes was co-created with the food service professionals from participating municipalities. The menu and recipes were modified to include more vegetables, fruits, berries, legumes, and sustainable fish, preferably from nearby sources, and less red meat and meat products, and adjusting milk consumption to the recommended level. In addition to the actual intervention, the intervention ECECs in one city in Päijät-Häme had recently introduced a new system of offering two different warm meal options for lunch, with one always being vegetarian. To support the acceptance of the new menu items, the intervention ECEC centres were provided with food education materials tailored to fit the menu changes. In addition, 2-hour training sessions to the early educators were organised in the beginning of the intervention and 1-hour training session at mid-point. In Finland, early childhood education and care (ECEC) is provided by professionals with varying levels of qualification, including ECEC teachers, ECEC social services professionals, childcare assistants, and special education teachers. Food education content was co-created in the workshop with ECEC professionals. In the third co-creation workshop with municipal decision makers, common goals and related challenges and opportunities were outlined to strengthen commitment to the intervention. The 10-month intervention started in the beginning of March 2022 and ended in the end of December 2022. During the intervention, parents in the intervention group were approached with an information letter detailing the planned modifications to the menu at ECEC and emphasizing that, as guardians, they play a pivotal role in supporting the development of a healthy and flexible relationship with food in their children. In addition, parents were granted access to the educational materials used in the intervention to further facilitate engagement with the intervention’s goals. The control ECEC centres did not change their practices during the intervention period. After the intervention they received all the materials and good practices tested in the intervention centres. Whether belonging to intervention or control ECEC centres could not be concealed from the personnel or the families.

### Recruitment

In the 23 ECEC centres participating in the study, all the families with a child aged 3–5 years attending full-time care, attending at least 80% of the weekly care hours, were invited to participate. Families were asked to complete questionnaires on the child’s dietary intake, background, and behavioral factors, and to collect the child’s stool sample, and bring the child to a clinic for blood sampling. Families received invitation and informed consent letters, which they returned to the ECEC centres. Recruitment was supported through Facebook and Instagram advertisements, a recruitment video, and flyers. Recruitment of the families took place between November 2021 and February 2022.

Recruitment of the families coincided with the COVID-19 pandemic, which initially prevented researchers from recruiting in person. Therefore, information sessions were arranged for families via Teams. Once restrictions eased, an additional recruitment round was conducted in February 2022, when researchers were allowed to visit all intervention ECEC centres and meet potential families outdoors. In total, 111 children (9% of those 1221 potentially invited) participated in the individual child-level assessment and formed the study sample (Table 1).

**Table 1:** Baseline characteristics of 111 study participants whose families consented with the study and provided background and/or dietary data^a^

	Intervention arm (*n* = 53)		Control arm (*n* = 58)		*p* value
	*n*	%	*n*	%	
*Sex*					0.463
Girl	26	49	31	53	
Boy	27	51	27	47	
*Age, years* ^b^					0.591
3–4	39	74	45	78	
5–6	14	26	13	22	
*Region*					0.828
Päijät-Häme	37	70	40	69	
South Ostrobothnia	16	30	18	31	
*Maternal education level* ^c^					0.035
Low	7	13	15	26	
Middle	19	36	28	48	
High	15	28	7	12	
Missing	12	23	8	14	
*Paternal education level* ^c^					0.965
Low	16	30	18	31	
Middle	18	34	21	36	
High	6	11	8	14	
Missing	13	25	11	19	
*Relative household income* ^d^					0.04
Lowest tertile (< 2100 €)	9	17	18	31	
Middle tertile (2100–2900 €)	9	17	18	31	
Highest tertile (> 2900 €)	17	32	10	17	
Missing	18	34	12	21	

^a^From five children with consent no background and/or diet data was obtained

^b^Age at the start of study

^c^Categories for education level: low: high school, vocational school or lower education; middle: bachelor’s degree or equivalent; high: master’s degree or higher education

^d^Gross household income, taking into account the number of people in the household

The project was implemented according to the prevailing national and EU legislation concerning medical research and data protection (488/1999; 1050/2018, GDPR) and international agreements (Helsinki Declaration). The ethics committee of the Hospital District of Helsinki and Uusimaa approved the study protocol on June 23, 2021. Municipals’ research permissions were obtained before the recruitment of the study in autumn 2021. Participating families gave written informed consents for their participation in the study.

### Dietary assessment methods

Children’s diet was assessed at baseline (before the intervention began), mid-term (follow-up I), and at the end of the intervention (follow-up II) by web-based food frequency questionnaires (FFQ) developed specifically for young children in the present study. The FFQs assessed children’s usual diet during the previous month. Separate FFQs were developed to measure food consumption at ECEC (ECEC-FFQ) and outside ECEC (home-FFQ). The ECEC-FFQs were completed by the ECEC professionals, and the home-FFQs were completed by the parents of the children.

The ECEC-FFQ was newly developed for this study. The FFQ contained questions on the portion size of the main lunch dishes, and on both frequency and portion size for other food items. The frequency of the main lunch dishes was not asked, as this information was obtained directly from the menu corresponding to each child’s municipality. The development incorporated investigation of food items typically served in Finnish ECECs based on openly available menus of municipal ECECs. The ECEC-FFQ comprised 27 main dish types (e.g. red meat stew, vegetable soup), 8 side dish types (e.g. pasta, potato), and 79 other food items. Pictures of dishes and food items within the FFQ facilitated individual portion size estimation.

Home-FFQ for young children was developed by modifying semiquantitative FFQ for women at the reproductive age [13]. The semiquantitative home-FFQ contained questions on the frequency of consumption of 142 food items and eight dietary supplement categories. Food items were selected to measure child’s total diet outside the ECEC, e.g., detailed questions of plant protein sources and fish species were included. Food record data from 3- to 6-year-old Finnish children from the Environmental Determinants of Diabetes in the Young (TEDDY) study and the Increased Health and Wellbeing in Preschools (DAGIS) study [8, 14] provided broad information on the diets of Finnish children guiding our selection of food items during the development of the home-FFQ. FFQ for food items consisted of frequencies of “not once”, “1–3 times per month”, “1–2 times per week”, “3–4 times per week”, “5–6 times per week”, “1–2 times per day”, “3–4 times per day” and “5 times or more per day” and for dietary supplement categories “not once”, “1–3 times per month”, “1–3 times per week”, “4–6 times per week”, “1 time per day”, “2 or more times per day”.

In order to calculate food consumption and nutrient intake, recipes representing the average content of each queried food/drink/supplement (FFQ recipes) were created and portion sizes were defined. For the ECEC-FFQ, FFQ-recipes for main meals were compiled based on the ECEC menus of the participating municipalities, with the menus from different municipalities weighted according to the number of participants from each municipality. The portion sizes for lunch meals and the portion sizes and intake frequencies queried in the ECEC-FFQ were used in calculations. For the home-FFQ, FFQ-recipes were compiled based on the food record data of 3-6-year-old Finnish children from the TEDDY study [14]. For each FFQ item, an average recipe was constructed based on the foods reported in the children’s food diaries from the TEDDY study. For foods and drinks with a portion size specified on the questionnaire, that portion size was used in calculations. For those without a specified portion size, the portion size was calculated based on the average portion sizes in the food records of the TEDDY study [14].

The data collected with the FFQs was further processed separately into dietary databases for calculating food consumption and nutrient intake. The frequency of each food/drink/supplement was converted to a daily usage frequency (e.g., 3–4 times per week = 3.5 / 7 = 0.5 per day) which was multiplied by portion size. This formed technical one-day food records for both the ECEC-FFQ and the home-FFQ.

The food consumption and nutrient intake calculations were performed with the in-house dietary calculation software Finessi at the Finnish Institute for Health and Welfare, which utilises the Finnish National Food Composition Database Fineli^®^ [15], ensuring that both FFQs based on an identical food classification structure. Dietary fibre calculation was based on new CODEX-compliant values [16]. To estimate food consumption at the raw ingredient level, the FFQ recipes were broken down into their constituent raw ingredients. Protein-containing ingredients were classified as either plant-based or animal-based. Animal-based ingredients were further classified into two groups: milk and meat (including fish and egg). This enabled separate calculations of plant, milk, and meat protein intake. Fish was classified into three categories based on the fish species and their typical origin on the Finnish market. The categories were: cultivated fish (rainbow trout, salmon, whitefish), fish from nearby areas (Baltic herring, roach, pike, vendace, perch, bream, flounder), and fish from elsewhere (saithe, tuna, cod). After calculating food consumption and nutrient intake, the data from the ECEC FFQ and home FFQ were combined to estimate total dietary intake.

### Assessment of the environmental impacts

The climate and biodiversity impacts were assessed using coefficients defined for the Finnish food consumption and derived from the Foodmin model [3, 4]. The model has been developed to simultaneously assess nutritional adequacy and climate impact of dietary scenarios [3], as well as agricultural land use and land-use-driven biodiversity impacts [4] of diets. The model consists of 91 different product groups following the food ingredient categorisation used in the Finnish National Food Composition Database Fineli^®^ [17]. The climate impact assessment in the model is based on life cycle assessment (LCA) information (CO2 eq.) on the 91 food product groups, both for domestic and imported, whereas the biodiversity impact assessment is based on agricultural land use related to the 91 product groups, following the grouping used in Fineli^®^ database, and their countries of origin when consumed in Finland. The biodiversity impact assessment was done by using the assessment method by Chaudhary and Brooks [18], which assesses the potentially disappeared fraction of species (PDF) due to land occupation and land use change. A more detailed description of the model and the climate impact assessment is presented in a previous publication by Saarinen et al. [3], and the biodiversity impact and land use assessment are presented in publication by Kyttä et al. [4]. To focus on diet quality rather than total intake, and to account for the fact that children’s energy consumption may have increased over the 10-month intervention period due to growth, the environmental impacts were energy-adjusted and analyzed per megajoule (MJ) of dietary intake.

### Statistical methods

Dietary and environmental measurements were taken at three different time points, and differences between intervention and control ECEC centres were tested over the period. We reported also means and standard deviation of measurements in all three time points. Multilevel linear mixed models were used with sex as fixed effects, and ID (subject code) and region of the ECEC centre as random effects. Due to rather small sample size of the study, the Kenward-Roger method was used in the analysis to improve the accuracy of tests and providing more precise p-values. Furthermore, the Bonferroni correction was used to address the problem of multiple comparisons, i.e. increased probability of a false positive finding by chance. We investigated the effects of the intervention on the outcomes by calculating effect sizes (Cohen’s d) for the observed changes in outcome values between the baseline and the end of follow-up measurements. The effect size was considered large if the lower bound of the confidence interval exceeded the conventional benchmark of 0.8.

All analysis were done by using the R statistical software [19].

## Results

**Fig. 1:**
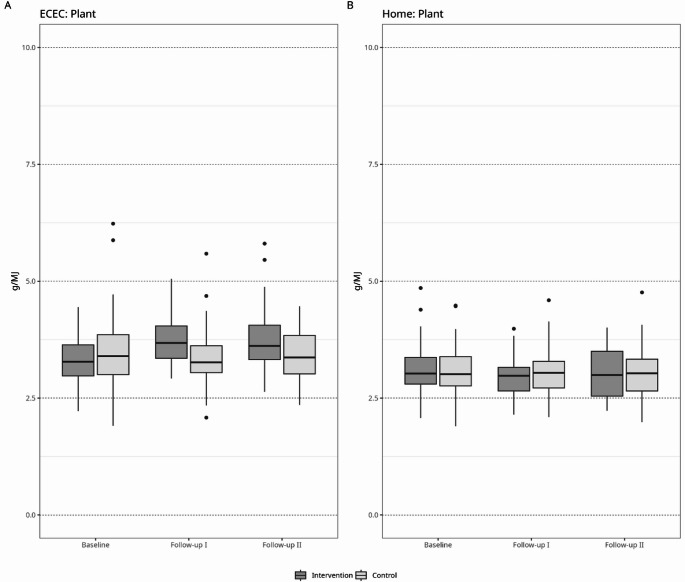
Plant protein (g/MJ) in the diet of 3-5-year-old Finnish children at the intervention and control arms of the FoodStep cluster-randomised intervention before the intervention (baseline), during the intervention (mid-intervention (follow-up I), and at the end (follow-up II)) **A** at early childhood education and care (ECEC) and **B** at home. Plant protein intake increased at ECEC by the intervention, *p* < 0.001

**Fig. 2:**
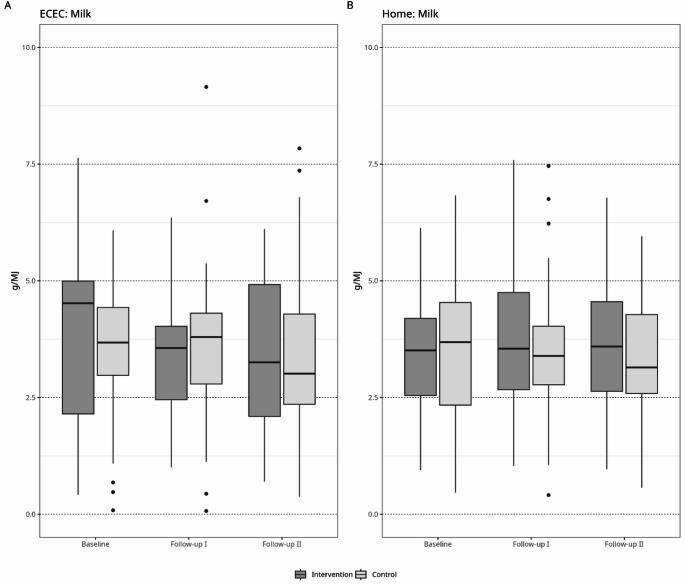
Milk protein (g/MJ) in the diet of 3-5-year-old Finnish children at the intervention and control arms of the FoodStep cluster-randomised intervention before the intervention (baseline), during the intervention (mid-intervention (follow-up I), and at the end of the intervention (follow-up II)) **A** at early childhood education and care (ECEC) and **B** at home. Milk protein intake did not change by the intervention

**Fig. 3:**
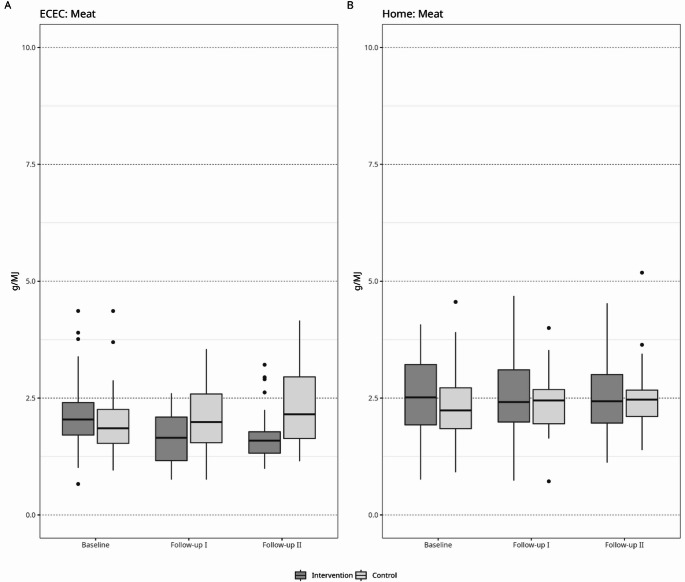
Meat protein (including fish and egg) (g/MJ) in the diet of 3-5-year-old Finnish children at the intervention and control arms of the FoodStep cluster-randomised intervention before (baseline), during (mid-intervention (follow-up I), and at the end of the intervention (follow-up II)) **A** at early childhood education and care (ECEC) and **B** at home. Meat protein intake decreased at ECEC by the intervention, *p* < 0.001

The baseline characteristics were similar among children in the intervention and control arms (Table 1). There was tendency to higher maternal education and family income in intervention arm, but differences did not bear multiple testing. Altogether, of the 111 study participants, the diet of 108 children (9% of the 1221 potentially invited) was assessed at baseline, 84 children (7%) at mid-term, and 68 children (6%) at the end of the intervention. At baseline, the children at the intervention and control ECEC centres had a similar diet both at ECEC and at home: no differences were detected in average food consumption, protein sources, energy, or nutrient intakes (Tables 2 and 3; Figs. 1, 2 and 3, Supplementary Tables 1–4).

**Table 2:** Daily food consumption (g/MJ) at early childhood education and care (ECEC) among 3-5-year-old Finnish children, FoodStep Study

Food consumption (g/MJ)	Baseline		Follow-Up I		Follow-Up II			*p* value for interaction^a, b^	
	Intervention *n* = 51	Control *n* = 57	Intervention *n* = 33	Control *n* = 51	Intervention *n* = 32		Control *n* = 36		
	Mean (SD)	Mean (SD)	Mean (SD)	Mean (SD)	Mean (SD)		Mean (SD)		
Vegetables (incl. mushrooms)	24.0 (10.2)	20.2 (10.4)	21.7 (7.34)	21.7 (11.2)	24.2 (11.0)		21.8 (6.69)	ns	
Cabbages	2.78 (1.75)	2.26 (1.54)	2.94 (1.23)	2.84 (1.88)	3.23 (1.70)		2.49 (1.01)	ns	
Roots (excl. potatoes)	7.24 (3.51)	6.48 (3.34)	7.64 (3.30)	7.09 (3.96)	7.32 (3.20)		6.88 (2.53)	ns	
Leaf vegetables	3.28 (1.74)	2.56 (1.62)	2.68 (1.01)	2.95 (1.76)	3.80 (2.45)		3.07 (1.61)	ns	
Fruit vegetables	6.74 (4.91)	5.74 (5.34)	6.19 (3.65)	6.01 (4.39)	7.61 (5.16)		5.96 (3.69)	ns	
Potatoes and potato products	14.9 (4.52)	13.2 (5.54)	15.8 (5.90)	13.7 (6.15)	15.8 (5.95)		17.0 (8.81)	ns	
Legumes and legume products	1.68 (1.03)	1.86 (1.61)	2.97 (1.47)	1.54 (0.79)	3.21 (1.39)		1.85 (0.96)	< 0.001	
Legume products (excl. soya)	0.12 (0.05)	0.11 (0.05)	0.21 (0.08)	0.11 (0.05)	0.24 (0.16)		0.13 (0.07)	0.001	
Soya products	0.06 (0.03)	0.07 (0.03)	0.09 (0.05)	0.08 (0.03)	0.09 (0.04)		0.08 (0.03)	ns	
Peas and beans	1.50 (1.00)	1.68 (1.59)	2.67 (1.42)	1.35 (0.78)	2.88 (1.23)		1.63 (0.92)	< 0.001	
Nuts and seeds	0.04 (0.02)	0.03 (0.01)	0.01 (0.00)	0.03 (0.01)	0.01 (0.00)		0.03 (0.01)	< 0.001	
Fruits and Berries	19.2 (9.18)	22.1 (12.6)	19.9 (8.18)	20.4 (10.9)	23.5 (8.98)		20.8 (12.0)	ns	
Fruits	16.3 (7.43)	19.0 (11.1)	16.2 (7.47)	17.6 (9.33)	19.2 (7.96)		18.1 (10.4)	ns	
Berries	2.90 (2.56)	3.09 (2.03)	3.71 (1.64)	2.82 (2.07)	4.34 (1.95)		2.75 (2.64)	ns	
Cereals	20.8 (4.06)	23.2 (6.83)	22.6 (3.89)	22.2 (5.32)	21.9 (5.61)		21.3 (4.09)	ns	
Oat	2.02 (0.86)	2.11 (1.00)	2.62 (0.95)	2.14 (0.73)	2.43 (1.32)		2.09 (0.73)	ns	
Rye	7.24 (3.51)	9.15 (5.37)	8.09 (2.38)	8.82 (5.13)	6.97 (3.19)		7.88 (4.02)	ns	
Barley	0.33 (0.20)	0.31 (0.20)	0.42 (0.25)	0.31 (0.18)	0.32 (0.23)		0.30 (0.17)	ns	
Wheat	9.28 (2.98)	9.68 (3.54)	9.73 (3.17)	8.89 (2.35)	10.3 (4.08)		8.81 (2.08)	ns	
Rice	1.42 (0.61)	1.38 (0.53)	0.99 (0.46)	1.44 (0.59)	1.12 (0.54)		1.61 (0.71)	ns	
Milk products	116 (65.4)	105 (41.8)	97.2 (44.2)	107 (48.1)	103 (54.3)		99.0 (55.4)	ns	
Cheese (fat > 17%)	0.35 (0.15)	0.25 (0.13)	0.37 (0.21)	0.24 (0.14)	0.41 (0.23)		0.29 (0.14)	ns	
Cheese (fat ≤ 17%)	0.68 (0.62)	0.73 (0.86)	0.87 (0.50)	0.77 (0.82)	0.65 (0.50)		0.84 (0.69)	ns	
Yoghurt and other sour milk products	8.45 (5.26)	8.68 (4.98)	9.60 (5.43)	8.64 (5.15)	9.54 (4.32)		9.47 (4.86)	ns	
Skimmed milk	64.0 (69.8)	56.7 (43.8)	43.5 (50.7)	65.3 (49.4)	56.3 (58.6)		57.0 (55.4)	ns	
Milk, containing fat	39.8 (48.1)	35.3 (44.6)	39.0 (39.2)	29.0 (35.1)	32.7 (34.4)		28.3 (32.9)	ns	
Plant based milk alternatives	8.55 (34.6)	3.90 (20.2)	4.33 (12.0)	3.98 (27.2)	4.64 (17.4)		9.57 (41.6)	ns	
Fats	7.14 (2.32)	7.53 (2.25)	7.89 (2.53)	7.59 (2.38)	7.62 (3.20)		7.47 (2.19)	ns	
Animal based fats	0.21 (0.09)	0.21 (0.08)	0.20 (0.08)	0.22 (0.11)	0.19 (0.11)		0.18 (0.07)	ns	
Vegetable based fats and oils	6.93 (2.34)	7.32 (2.24)	7.69 (2.53)	7.38 (2.41)	7.44 (3.24)		7.30 (2.21)	ns	
Fish and shellfish	2.78 (1.40)	2.28 (1.10)	2.07 (1.15)	2.22 (1.03)	2.09 (0.91)		2.81 (1.26)	ns	
Cultivated fish	0.71 (0.33)	0.62 (0.28)	0.54 (0.29)	0.63 (0.28)	0.54 (0.20)		0.79 (0.34)	ns	
Fish from nearby area	0.01 (0.01)	0.01 (0.01)	0.23 (0.19)	0.01 (0.01)	0.19 (0.12)		0.02 (0.01)	< 0.001	
Fish from elsewhere	2.05 (1.11)	1.64 (0.85)	1.30 (0.82)	1.58 (0.80)	1.35 (0.77)		2.01 (0.95)	0.027	
Meat	7.76 (2.75)	7.26 (2.35)	5.68 (2.19)	7.71 (2.57)	5.63 (2.60)		8.61 (3.31)	< 0.001	
Red meat, total	2.77 (1.05)	2.43 (1.00)	1.69 (0.66)	2.47 (0.89)	1.60 (0.74)		3.08 (1.58)	< 0.001	
Beef	1.84 (0.72)	1.57 (0.65)	0.94 (0.46)	1.58 (0.57)	1.02 (0.64)		2.02 (1.11)	< 0.001	
Pork	0.88 (0.36)	0.80 (0.36)	0.63 (0.33)	0.83 (0.32)	0.53 (0.22)		1.00 (0.52)	< 0.001	
Poultry	2.48 (0.97)	2.30 (1.00)	2.42 (1.17)	2.36 (0.93)	2.41 (1.05)		2.88 (1.17)	ns	
Sausages and meat cuts	2.50 (1.24)	2.54 (1.22)	1.58 (0.85)	2.89 (1.76)	1.62 (1.19)		2.64 (1.22)	0.001	
Eggs	1.86 (1.16)	1.70 (1.01)	1.80 (0.85)	2.38 (1.72)	2.16 (1.03)		2.19 (1.21)	ns	
Sugar and sweets	2.33 (1.02)	2.80 (1.66)	2.39 (1.01)	2.87 (1.76)	2.50 (0.97)		2.10 (0.72)	ns	
Drinks	85.1 (58.5)	74.1 (31.2)	107 (55.7)	95.2 (44.12)	87.2 (65.2)		107 (76.9)	ns	
Sugar-sweetened soft drinks	0.00 (0.00)	0.00 (0.00)	0.00 (0.00)	0.00 (0.00)	0.00 (0.00)		0.00 (0.00)	ns	
Artificially sweetened soft drinks	0.00 (0.00)	0.00 (0.00)	0.00 (0.00)	0.00 (0.00)	0.00 (0.00)		0.00 (0.00)	ns	
Juice drinks	0.93 (1.55)	0.67 (1.57)	1.08 (1.99)	0.53 (1.20)	0.33 (0.82)		0.51 (1.43)	ns	
Juices	0.90 (1.15)	0.74 (0.65)	0.79 (0.89)	0.59 (0.28)	0.70 (0.35)		0.76 (0.65)	ns	

^a^Bonferroni-corrected *p* values, multilevel linear mixed models were used with sex as fixed effects, and ID and region of the ECEC centre as random effects

^b^Not significant (ns)

**Table 3:** Daily consumption (g/MJ) at home among 3-5-year-old finnish children, FoodStep study

Food consumption (g/MJ)	Baseline		Follow-Up I		Follow-Up II			*p* value for interaction^a,b^	
	Intervention *n* = 29	Control *n* = 47	Intervention *n* = 33	Control *n* = 39	Intervention *n* = 31		Control *n* = 37		
	Mean (SD)	Mean (SD)	Mean (SD)	Mean (SD)	Mean (SD)		Mean (SD)		
Vegetables (incl. mushrooms)	19.7 (9.03)	19.0 (9.07)	22.0 (12.4)	19.3 (9.14)	18.6 (9.50)		18.0 (9.65)	ns	
Cabbages	1.18 (1.35)	0.83 (0.86)	1.16 (1.22)	0.69 (0.60)	1.06 (1.41)		0.67 (0.79)	ns	
Roots (excl. potatoes)	4.47 (2.41)	6.27 (4.32)	4.50 (3.30)	5.66 (3.73)	5.64 (5.47)		5.66 (3.91)	ns	
Leaf vegetables	1.55 (1.21)	1.42 (1.20)	1.76 (1.89)	1.20 (1.01)	1.47 (1.46)		1.00 (0.82)	ns	
Fruit vegetables	10.1 (6.70)	8.43 (5.38)	12.1 (9.68)	9.72 (6.38)	8.14 (4.71)		8.65 (6.50)	ns	
Potatoes and potato products	10.7 (3.19)	10.1 (4.72)	10.3 (3.24)	11.0 (5.93)	10.0 (2.79)		10.1 (4.05)	ns	
Legumes and legume products	2.33 (1.91)	1.79 (1.56)	2.11 (1.70)	1.41 (1.25)	2.26 (2.03)		1.38 (1.48)	ns	
Legume products (excl. soya)	0.24 (0.38)	0.09 (0.19)	0.19 (0.32)	0.06 (0.13)	0.33 (0.66)		0.09 (0.20)	ns	
Soya products	0.11 (0.22)	0.05 (0.16)	0.08 (0.21)	0.05 (0.16)	0.13 (0.26)		0.06 (0.14)	ns	
Peas and beans	1.98 (1.74)	1.65 (1.40)	1.83 (1.52)	1.29 (1.17)	1.80 (1.60)		1.22 (1.33)	ns	
Nuts and seeds	0.56 (0.70)	0.59 (0.86)	0.57 (0.59)	0.42 (0.62)	0.53 (0.50)		0.48 (0.56)	ns	
Fruits and Berries	35.9 (17.8)	43.3 (22.1)	36.0 (16.4)	39.6 (19.0)	38.0 (22.5)		43.7 (20.6)	ns	
Fruits	31.7 (17.5)	38.5 (20.9)	31.2 (16.1)	34.2 (17.1)	33.6 (22.5)		38.7 (18.4)	ns	
Berries	4.13 (3.78)	4.77 (4.09)	4.82 (4.35)	5.35 (6.15)	4.41 (3.26)		5.03 (4.55)	ns	
Cereals	15.5 (3.08)	16.0 (4.23)	15.0 (3.34)	16.2 (3.09)	15.5 (3.94)		16.4 (3.03)	ns	
Oat	1.58 (1.03)	2.41 (2.86)	1.74 (1.25)	2.01 (1.70)	1.63 (1.28)		1.96 (1.49)	ns	
Rye	2.70 (1.54)	2.67 (1.90)	2.36 (1.31)	2.56 (1.84)	2.59 (1.35)		2.83 (2.46)	ns	
Barley	0.21 (0.28)	0.31 (0.54)	0.24 (0.29)	0.30 (0.59)	0.18 (0.24)		0.14 (0.12)	ns	
Wheat	8.10 (1.94)	7.61 (2.38)	7.69 (1.94)	8.19 (1.93)	7.95 (2.59)		8.25 (1.68)	ns	
Rice	1.99 (0.79)	2.14 (0.92)	2.02 (0.64)	2.15 (1.02)	2.14 (0.83)		2.22 (0.92)	ns	
Milk products	84.8 (40.4)	91.1 (42.1)	92.3 (47.0)	90.3 (44.8)	92.9 (45.7)		81.3 (33.7)	ns	
Cheese (fat > 17%)	1.65 (1.41)	1.44 (1.27)	1.71 (1.49)	1.50 (1.11)	1.54 (1.34)		1.45 (1.46)	ns	
Cheese (fat ≤ 17%)	1.46 (1.70)	1.17 (1.46)	1.41 (1.60)	1.23 (1.61)	1.23 (1.25)		1.25 (1.60)	ns	
Yoghurt and other sour milk products	21.6 (14.7)	25.1 (15.6)	25.6 (18.5)	24.8 (15.3)	25.1 (19.4)		26.2 (15.7)	ns	
Skimmed milk	18.7 (27.7)	11.7 (17.2)	23.8 (37.3)	18.1 (30.5)	24.0 (28.4)		11.7 (16.4)	ns	
Milk, containing fat	38.6 (36.4)	49.0 (41.7)	35.8 (37.0)	41.1 (35.5)	38.1 (38.4)		37.8 (29.2)	ns	
Plant based milk alternatives	13.8 (28.9)	11.0 (28.5)	9.04 (29.1)	11.1 (31.8)	8.16 (24.1)		8.77 (29.7)	ns	
Fats	4.29 (1.56)	3.59 (1.26)	3.95 (1.11)	3.45 (0.98)	3.67 (1.13)		3.63 (1.00)	ns	
Animal based fats	1.69 (1.49)	1.37 (1.29)	1.27 (1.05)	1.23 (0.87)	0.99 (0.68)		1.33 (1.28)	ns	
Vegetable based fats and oils	2.60 (1.30)	2.22 (1.07)	2.68 (1.30)	2.21 (1.20)	2.68 (1.36)		2.30 (1.05)	ns	
Fish and shellfish	2.05 (1.43)	1.91 (0.86)	2.40 (1.64)	1.83 (0.88)	2.27 (1.22)		1.89 (1.27)	ns	
Cultivated fish	1.06 (0.82)	1.15 (0.60)	1.33 (0.99)	0.94 (0.66)	1.26 (0.89)		1.08 (0.72)	ns	
Fish from near-by area	0.42 (0.91)	0.18 (0.35)	0.52 (0.72)	0.14 (0.27)	0.59 (0.78)		0.18 (0.41)	ns	
Fish from elsewhere	0.40 (0.55)	0.48 (0.49)	0.42 (0.47)	0.65 (0.58)	0.27 (0.33)		0.54 (0.59)	ns	
Meat	10.2 (4.34)	9.14 (4.00)	9.56 (4.68)	9.70 (3.07)	9.46 (4.73)		9.84 (3.67)	ns	
Red meat, total	3.80 (1.89)	3.97 (2.87)	3.89 (2.79)	3.89 (2.09)	3.35 (1.83)		3.90 (2.53)	ns	
Beef	1.87 (1.36)	2.61 (2.65)	2.07 (1.73)	2.48 (1.93)	1.46 (1.11)		2.47 (1.88)	ns	
Pork	1.32 (1.29)	0.98 (1.02)	1.32 (1.26)	1.16 (1.06)	1.35 (1.49)		1.00 (0.96)	ns	
Poultry	3.52 (2.85)	2.47 (1.48)	2.64 (1.65)	2.52 (1.57)	3.00 (2.13)		2.74 (1.52)	ns	
Sausages and meat cuts	2.88 (1.53)	2.70 (1.25)	3.04 (1.95)	3.29 (1.45)	3.11 (2.67)		3.20 (1.56)	ns	
Eggs	2.83 (1.42)	2.82 (1.65)	2.97 (1.80)	2.81 (2.01)	3.42 (2.78)		2.98 (1.57)	ns	
Sugar and sweets	5.63 (2.16)	4.81 (2.37)	4.94 (2.26)	4.45 (2.06)	5.57 (2.43)		5.32 (2.27)	ns	
Drinks	121 (52.9)	117 (52.7)	133 (53.7)	122 (65.9)	134 (51.4)		129 (52.3)	ns	
Sugar-sweetened soft drinks	2.36 (3.72)	1.29 (2.33)	1.31 (2.24)	1.28 (2.79)	2.08 (3.60)		1.48 (2.11)	ns	
Artificially sweetened soft drinks	0.63 (1.48)	1.18 (3.21)	1.30 (2.53)	0.89 (2.11)	1.48 (3.03)		1.26 (2.64)	ns	
Juice drinks	7.17 (14.6)	4.91 (5.66)	10.7 (20.4)	6.57 (8.32)	11.2 (26.6)		5.51 (5.23)	ns	
Juices	3.94 (4.89)	3.34 (3.59)	5.51 (8.95)	3.26 (3.56)	4.35 (5.79)		3.17 (3.80)	ns	

^a^Bonferroni-corrected *p* values, multilevel linear mixed models were used with sex as fixed effects, and ID and region of the ECEC centre as random effects

^b^Not significant (ns)

At ECEC the intervention increased the consumption of legumes and legume products, while the consumption of nuts and seeds decreased. The consumption of fish from nearby areas increased while that from other areas decreased. Also, at ECEC, the consumption of red meat, sausages, and other meat products decreased by the intervention (Table 2). The intake of plant protein increased, while that of meat protein i.e., animal protein from meat and meat products, egg, and fish decreased at the intervention ECEC. The intervention did not affect berries and fruit consumption (Table 2) or milk protein intake (Figs. 1, 2 and 3a). At home, no changes in food consumption or protein intake from plant, meat, or milk products were detected (Table 3; Figs. 1, 2 and 3b). Changes in food consumption by intervention were similar in girls and boys.

The effects of the intervention were also evaluated by effect sizes. Effect sizes were large for increases in fish from nearby areas (effect size 2.54), legumes and legume products (1.79), legume products excluding soya (1.70), peas and beans (1.67), soya products (1.00), and for decreases in nuts and seeds (effect size 2.69), beef (1.36), red meat, game and offal (1.31), pork (1.07), meat including red meat, poultry, sausages, offal (0.96), fish from elsewhere (0.93), sausages and meat cuts (0.9).

The energy intake did not change by the intervention. Of nutrients only the intake of cholesterol and selenium decreased by the intervention at ECEC centres (Supplementary Tables 1 and 2). The energy and nutrient intake at home did not change by the intervention. Children’s total diet at ECEC centres and home is presented in Table 4. The diets at ECEC and home were similar regarding the intake of all energy adjusted nutrients studied (data not shown). At baseline, food eaten at ECEC provided on average 37% of children’s energy intake, while home diet contributed 63%. At the end of the intervention, these proportions were 32% and 68%, respectively.

**Table 4:** Daily intake of energy and macronutrients (mean and standard deviation) among 3-5-year-old finnish children, FoodStep study

	Baseline		Follow-Up I		Follow-Up II	
	Intervention *n* = 28	Control *n* = 46	Intervention *n* = 27	Control *n* = 36	Intervention *n* = 23	Control *n* = 28
	Mean (SD)	Mean (SD)	Mean (SD)	Mean (SD)	Mean (SD)	Mean (SD)
Energy (MJ)	7.82 (1.74)	8.22 (1.46)	7.77 (1.90)	7.45 (1.45)	7.46 (2.08)	7.68 (1.95)
Protein (%E)	15.4 (1.48)	15.3 (1.48)	15.6 (1.62)	15.2 (1.62)	15.5 (1.80)	15.1 (1.47)
Fat (%E)	32.0 (3.39)	30.6 (2.97)	32.0 (3.04)	30.4 (2.98)	31.4 (3.86)	30.7 (2.35)
SFA (%E)	12.1 (1.78)	11.7 (1.60)	11.9 (1.29)	11.6 (1.52)	11.5 (1.66)	11.6 (1.50)
MUFA (%E)	11.4 (1.34)	10.8 (1.37)	11.5 (1.47)	10.8 (1.39)	11.3 (1.78)	10.9 (1.21)
PUFA (%E)	5.48 (0.87)	5.16 (0.90)	5.57 (0.89)	5.11 (0.88)	5.67 (1.14)	5.23 (0.94)
n-3 (%E)	1.33 (0.27)	1.23 (0.23)	1.38 (0.27)	1.21 (0.24)	1.42 (0.33)	1.27 (0.23)
n-6 (%E)	4.04 (0.64)	3.83 (0.70)	4.08 (0.65)	3.81 (0.68)	4.16 (0.83)	3.87 (0.76)
Carbohydrates (%E)	50.2 (2.88)	51.6 (3.00)	50.1 (2.67)	52.0 (2.99)	50.9 (3.49)	51.7 (2.61)
Sucrose (%E)	10.1 (2.37)	10.0 (2.47)	9.66 (2.12)	10.2 (2.68)	10.1 (2.15)	10.4 (1.94)
Dietary fibre (g/MJ)	3.73 (0.66)	3.89 (0.72)	3.64 (0.48)	3.77 (0.53)	3.58 (0.50)	3.88 (0.54)

## Environmental impacts

**Fig. 4:**
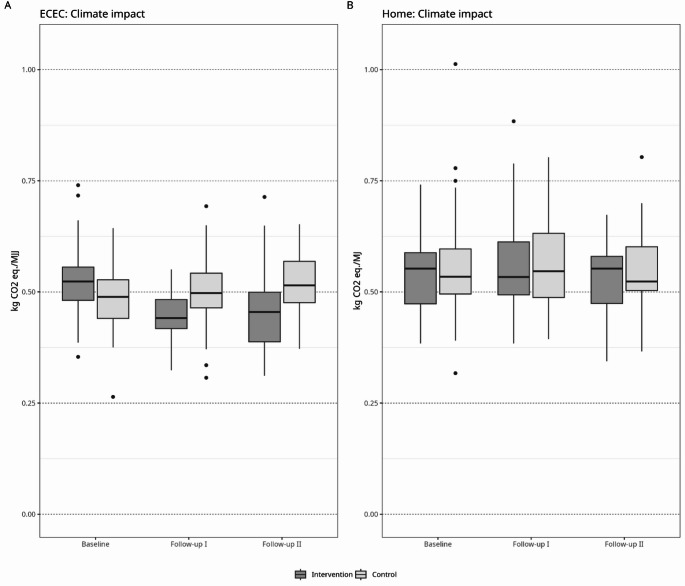
Climate impact of food (kg CO2 eq./MJ) at the intervention and control arm of the FoodStep cluster-randomised intervention before (baseline), during (mid-intervention (follow-up I), and at the end of the intervention (follow-up II)) **A** at early childhood education and care (ECEC) and **B** home. Climate impact of food (kg CO2 eq./MJ) decreased at ECEC (A) by the intervention, *p* < 0.001

**Fig. 5:**
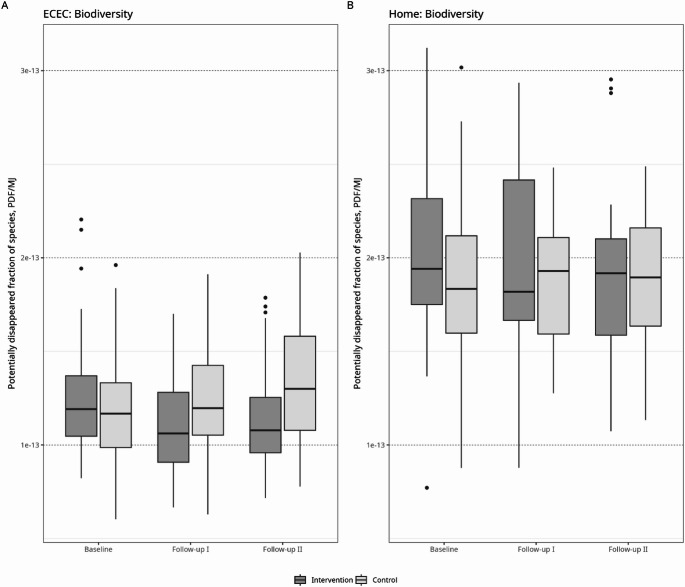
Biodiversity impact of food (PDF/MJ) at the intervention and control arm of the FoodStep cluster-randomised intervention before (baseline), during the intervention (mid-intervention (follow-up I), and at the end of the intervention (follow-up II)) **A** at early childhood education and care (ECEC) and **B** at home. Biodiversity impact of food (PDF/MJ) decreased at ECEC (A) by the intervention, *p* = 0.003

At the baseline the children at the intervention and control ECEC centres had a similar diet both at ECEC and at home: no differences were detected in climate impacts (kg CO2 eq./MJ) or biodiversity impacts of food (PDF/MJ). Climate impacts of food decreased at the intervention ECEC compared to the control ECEC (*p* < 0.001, Fig. 4), the change between baseline and follow-up II being − 11.3%. Also, biodiversity impacts of food decreased at the intervention ECEC compared to the control ECEC (*p* = 0.003, Fig. 5), the percentage change between baseline and follow-up II being − 9.6%. At home, no changes in the climate impact or biodiversity impact were observed.

## Discussion

This study showed that systemic change in menus and food education at ECEC resulted in healthier diet with reduced environmental impacts among children. The consumption of legumes, legume products, and fish from nearby areas increased and that of red meat and processed meat decreased among children during the 10-month intervention. These changes had large effect sizes, indicating substantial practical impact. Beneficial changes in the climate and biodiversity impacts were observed. No harmful effects were observed in nutrient intake. However, there was no moderation in milk consumption or increase in berry and fruit consumption.

Assessing the effects of the intervention on child’s individual food consumption and nutrient intake as well as on climate and biodiversity impacts at the same time is a unique strength of this study. Co-creation in workshops with the food service and ECEC professionals and municipal decision makers were insightful, common goals and related challenges and opportunities were outlined to strengthen commitment to the intervention. The modified menu was implemented without observed decreases in protein, vitamin or mineral intakes, with the exception of selenium. The use of nuts and seeds was very low (less than 0.1 g/day at ECEC) at both the baseline and end of the intervention and thus unlikely to influence health or environmental outcome, although there was a significant difference. The decrease in selenium intake was small, and the mean intake remained sufficient. These changes in food consumption were in alignment with the existing dietary guidelines for ECEC [12]. Enhanced food education supported both the implementation and the acceptability of the modified menus [20]. The changes in food consumption observed speak in favour of good acceptability among children. The length of intervention showed to be sufficient for dietary change, but longer intervention would been needed to study the sustainability of the change.

A weakness of the study was that the recruitment of the families coincided with COVID-19 pandemic, which prevented researchers from recruiting in person and could have affected participation. Also, response rate declined towards the end of the study, so the size of the study population is relatively small and may not fully represent the target population. The low participation rate of the families does not compromise the comparability of the intervention and control arms within the intervention design, but it affects to whom the results can be applied. However, for ethical reasons we were not allowed to study non-participants i.e., to evaluate the selection process. Also, family selection is unlikely to influence changes within the ECEC centres. Additional weakness of the study was that randomisation could not be performed in the South Ostrobothnia ECEC centres for practical reasons. FFQs were developed for the present study based on previous studies [8, 13, 14] but not validated for this age group. Even though FFQ is not considered appropriate for estimating actual absolute food and nutrient intake, it can be used for categorising persons’ food consumption and nutrient intake [13], and for evaluating change in food consumption [21]. The main aim of this study was not to try to change the children’s diet at home. The current study included only minor involvement of the parents. An umbrella review by Matwiejczyk et al. [22] summarised that positive outcomes of interventions promoting healthy eating can be further strengthened with parental involvement and engagement. Multi-level, multi-component strategies are recommended and were used also in the current study.

Previous ECEC-based healthy eating interventions focusing on the entire diet with assessing the total food consumption alongside nutritional and environmental impacts in preschool children are scarce. 15% reduction in carbon footprint was observed in food service level in Danish daycare study [23] which was a bit higher than 11% reduction observed in the current study. Systemic menu-level interventions or modelling approaches to reduce the climate impact of the provision of food to children have been implemented in school environments [24, 25]. Nutritional quality was retained by partial replacement of meat-based dishes with plant-based dishes in Swedish school meals [24]. Overall, changes in ECEC- and school-based interventions remain small [26, 27]. The present study found that children’s diet shifted towards healthier and more environmentally sustainable food consumption without compromising nutrient intake, thereby strengthening the existing positive evidence base. The Finnish ECEC system provides daily meals to all children, which is not common elsewhere and may help in introducing dietary chances.

The climate impacts observed in this study, around 0.5 kg CO_2_ eq/MJ, were somewhat higher than those reported in previous research, being around 0.4 kg CO_2_ eq/MJ in a Norwegian dietary study involving 2-year-old children and in a Swedish dietary study on 4-year-old children [28, 29]. However, the system boundaries of this study included the impacts from cradle to plate, whereas the other studies did not consider the impact from food preparation. For biodiversity impacts, direct comparisons with other studies were not feasible due to methodological differences in biodiversity impact assessment. In our study, both carbon footprint and biodiversity impact of food were higher for home than ECEC food. To achieve more systemic change, the adaptation of changes in consumption outside of ECEC should also be supported.

Following the approach of Jacobsen et al. [28] and using the planetary boundaries defined for food system by the EAT-Lancet Commission [1] as a benchmark, the climate impacts observed in this study remained above the planetary boundaries threshold even after the dietary intervention. This finding indicates that more extreme changes would be needed to achieve sustainable diets in terms of climate change.

ECEC is an ideal setting for systemic change in Finland, since nearly all children participate in it [10] enabling comprehensive food education and daily meals provided by food services [11, 12]. Even small dietary changes can have a meaningful impact at the population level, and familiarisation with new foods and dishes in ECEC may also influence children’s food preferences beyond the ECEC.

Although adapting to new foods and dishes can be challenging for children, ECEC settings provide a safe and supportive environment for such exposure [11, 12]. The new menu items were well received in the present study, as the amount of food waste did not increase despite the menu changes [30].

To conclude, this study demonstrated that well-planned systemic improvements in meal provision and food education within ECEC can promote healthier and more climate-friendly diets among children without compromising adequate nutrient intake. However, larger-scale interventions with extended follow-up are necessary to evaluate long-term sustainability and broader generalizability of these findings.

## Supplementary Information

Below is the link to the electronic supplementary material.

Supplementary Material 1

## Data Availability

Researchers interested in the data from this study may contact principal investigator Suvi M. Virtanen, suvi.virtanen@tuni.fi.

## Declarations

### Clinical trial registry

NCT05249946; registration date: 2021-11-29.
