# Supplementary material for: The effect of system-level intervention in early childhood education and care on children’s dietary intake and environmental impact of the diet
Source: Eur J Nutr. 2026 Jul 17;65(5):216. doi: 10.1007/s00394-026-04064-x (PMC13379481; doi:10.1007/s00394-026-04064-x)
Supplement: Supplementary file 1 — Supplementary Material 1 [file 394_2026_4064_MOESM1_ESM.pdf]

Supplementary Material to

The effect of system-level intervention in early childhood education and care on children's dietary intake and environmental impact of the diet

Mari Åkerlund, Venla Kyttä, Jelena Meinilä, Arto Pietilä, Tuuli E. Korhonen, Henna Vepsäläinen, Liisa Korkalo, Satu Kinnunen, Sari Niinistö, Susanna Raulio, Leena Forma, Maijaliisa Erkkola, Merja Saarinen, Suvi M. Virtanen

Corresponding Authors:

MÅ and VK share the first authorship.

Mari Åkerlund, Department of Public Health, Finnish Institute for Health and Welfare, P.O. Box 30, FI-00271 Helsinki, Finland, E-mail mari.akerlund@thl.fi

Venla Kyttä, Natural Resources Institute Finland, Latokartanonkaari 9, FI-00790 Helsinki, Finland, E-mail venla.kytta@luke.fi

Supplementary Tables

Supplementary Table 1 Daily intake of energy and macronutrients (mean and standard deviation) at ECEC among 3-5-year-old Finnish children, FoodStep Study

Supplementary Table 2 Daily intake of selected nutrients (mean and standard deviation) at ECEC among 3-5-year-old Finnish children, FoodStep Study

Supplementary Table 3 Daily intake of energy and macronutrients (mean and standard deviation) at home among 3-5-year-old Finnish children, FoodStep Study

Supplementary Table 4 Daily intake of selected nutrients (mean and standard deviation) at home among 3-5-year-old Finnish children, FoodStep Study

Supplementary Table 1 Daily intake of energy and macronutrients (mean and standard deviation) at ECEC among 3-5-year-old Finnish children, FoodStep Study

	Baseline		Follow-Up I		Follow-Up II		p value for interaction[a,b]
	Intervention n = 51	Control n = 57	Intervention n = 33	Control n = 51	Intervention n = 32	Control n = 36	
	Mean (SD)	Mean (SD)	Mean (SD)	Mean (SD)	Mean (SD)	Mean (SD)	
Energy (MJ)	2.40 (1.00)	2.40 (0.90)	2.66 (1.04)	2.16 (0.84)	2.61 (1.03)	2.37 (0.84)	ns
Protein (g/MJ)	9.27 (1.61)	8.86 (1.08)	8.66 (1.05)	9.01 (1.20)	8.86 (1.56)	9.10 (1.45)	ns
Protein (%E)	15.8 (2.73)	15.1 (1.83)	14.7 (1.78)	15.3 (2.05)	15.1 (2.65)	15.5 (2.47)	ns
Fat (g/MJ)	8.92 (1.46)	8.91 (1.32)	9.24 (1.69)	9.08 (1.20)	8.90 (1.86)	9.08 (1.17)	ns
Fat (%E)	33.0 (5.40)	33.0 (4.87)	34.2 (6.25)	33.6 (4.43)	32.9 (6.88)	33.6 (4.33)	ns
SFA (%E)	11.4 (2.15)	11.3 (2.00)	11.7 (2.41)	11.5 (1.77)	11.0 (2.22)	11.3 (1.85)	ns
MUFA (%E)	12.7 (2.38)	12.7 (2.20)	13.1 (2.66)	13.0 (2.04)	12.7 (3.12)	13.1 (1.92)	ns
PUFA (%E)	6.68 (1.38)	6.79 (1.21)	7.11 (1.41)	6.87 (1.26)	6.95 (1.79)	6.98 (1.12)	ns
n-3 (%E)	1.79 (0.43)	1.82 (0.38)	1.93 (0.46)	1.84 (0.39)	1.90 (0.57)	1.88 (0.35)	ns
n-6 (%E)	4.83 (0.96)	4.91 (0.84)	5.12 (0.95)	4.98 (0.88)	5.00 (1.23)	5.04 (0.80)	ns
Cholesterol (g/MJ)	20.0 (5.15)	18.4 (5.83)	16.8 (3.97)	21.1 (8.27)	17.5 (3.85)	20.7 (6.10)	0.019
Carbohydrates (g/MJ)	28.2 (2.28)	28.5 (2.32)	28.0 (3.01)	28.0 (1.96)	28.5 (2.96)	27.9 (2.09)	ns
Carbohydrates (%E)	48.7 (3.90)	49.2 (3.91)	48.3 (5.17)	48.4 (3.37)	49.3 (5.09)	48.2 (3.51)	ns
Sucrose (g/MJ)	3.51 (1.58)	3.65 (1.38)	3.42 (1.21)	3.64 (1.38)	3.43 (1.07)	3.51 (1.63)	ns
Sucrose (%E)	5.97 (2.69)	6.21 (2.35)	5.81 (2.06)	6.18 (2.34)	5.83 (1.82)	5.96 (2.77)	ns
Dietary fibre (g/MJ)	3.90 (0.72)	4.18 (0.90)	4.23 (0.54)	4.06 (0.90)	4.18 (0.83)	4.07 (0.90)	ns
IDF (g/MJ)	2.28 (0.45)	2.43 (0.50)	2.49 (0.33)	2.37 (0.55)	2.50 (0.51)	2.37 (0.57)	ns
SDFP (g/MJ)	1.00 (0.17)	1.03 (0.18)	1.07 (0.15)	1.02 (0.18)	1.05 (0.19)	1.06 (0.20)	ns
SDFS (g/MJ)	0.57 (0.14)	0.65 (0.24)	0.62 (0.09)	0.62 (0.22)	0.58 (0.15)	0.60 (0.18)	ns

a) Bonferroni-corrected p values, multilevel linear mixed models were used with sex as fixed effects, and ID and region of the ECEC centre as random effects

b) Not significant (ns)

Supplementary Table 2 Daily intake of selected nutrients (mean and standard deviation) at ECEC among 3-5-year-old Finnish children, FoodStep Study

	Baseline		Follow-Up I		Follow-Up II		p value for interaction[a,b]
	**Intervention**<br><br>**n = 51**	**Control**<br><br>**n = 57**	**Intervention**<br><br>**n = 33**	**Control**<br><br>**n = 51**	**Intervention**<br><br>**n = 32**	**Control**<br><br>**n = 36**	
	**Mean (SD)**	**Mean (SD)**	**Mean (SD)**	**Mean (SD)**	**Mean (SD)**	**Mean (SD)**	
Vitamin A (µg RAE/MJ)	124 (33.0)	119 (26.0)	133 (32.3)	126 (26.9)	127 (32.7)	124 (20.8)	ns
Vitamin C (mg/MJ)	11.6 (3.80)	11.4 (5.24)	11.0 (2.84)	11.6 (5.18)	12.5 (4.26)	11.7 (4.15)	ns
Vitamin D (µg/MJ)	2.45 (0.62)	2.39 (0.55)	2.34 (0.58)	2.44 (0.54)	2.34 (0.66)	2.38 (0.56)	ns
Vitamin E (mg/MJ)	1.35 (0.25)	1.36 (0.19)	1.40 (0.22)	1.38 (0.20)	1.40 (0.30)	1.39 (0.19)	ns
Thiamin (mg/MJ)	0.14 (0.02)	0.14 (0.02)	0.14 (0.02)	0.14 (0.02)	0.14 (0.02)	0.14 (0.02)	ns
Riboflavin (mg/MJ)	0.29 (0.10)	0.27 (0.07)	0.25 (0.08)	0.28 (0.08)	0.27 (0.09)	0.27 (0.09)	ns
Niacin (mg/MJ)	3.00 (0.44)	2.85 (0.36)	2.76 (0.31)	2.88 (0.36)	2.83 (0.48)	2.95 (0.45)	ns
Vitamin B6 (mg/MJ)	0.21 (0.03)	0.20 (0.03)	0.19 (0.02)	0.20 (0.03)	0.20 (0.03)	0.21 (0.04)	ns
Vitamin B12 (mg/MJ)	0.64 (0.24)	0.57 (0.16)	0.53 (0.16)	0.60 (0.19)	0.55 (0.22)	0.58 (0.21)	ns
Folate (µg/MJ)	29.6 (4.05)	28.8 (5.09)	30.7 (4.11)	29.4 (5.34)	32.2 (5.67)	29.6 (4.37)	ns
Potassium (mg/MJ)	470 (80.5)	442 (81.5)	444 (66.8)	450 (81.0)	465 (86.4)	454 (78.7)	ns
Calcium (mg/MJ)	183 (69.4)	163 (44.9)	157 (47.4)	168 (54.9)	165 (60.7)	166 (62.0)	ns
Magnesium (mg/MJ)	45.5 (5.78)	45.2 (5.62)	45.6 (4.90)	45.2 (4.94)	45.7 (5.51)	45.3 (4.78)	ns
Salt (g/MJ)	0.89 (0.09)	0.87 (0.10)	0.90 (0.08)	0.88 (0.11)	0.88 (0.08)	0.90 (0.10)	ns
Iron (mg/MJ)	1.23 (0.17)	1.25 (0.19)	1.30 (0.14)	1.24 (0.17)	1.29 (0.18)	1.28 (0.19)	ns
Selenium (µg/MJ)	8.59 (1.40)	8.00 (1.14)	7.65 (0.99)	8.36 (1.32)	7.91 (1.37)	8.38 (1.41)	0.028
Phosphorus (mg/MJ)	214 (47.8)	205 (29.5)	202 (33.0)	208 (33.0)	205 (40.3)	204 (37.2)	ns
Iodine (µg/MJ)	34.0 (8.25)	31.7 (5.55)	31.7 (5.53)	32.0 (6.31)	32.3 (6.87)	32.0 (7.33)	ns
Zinc (mg/MJ)	1.41 (0.22)	1.38 (0.17)	1.34 (0.16)	1.39 (0.15)	1.34 (0.20)	1.39 (0.16)	ns

[a)] Bonferroni-corrected p values, multilevel linear mixed models were used with sex as fixed effects, and ID and region of the ECEC centre as random effects

[b)] Not significant (ns)

Supplementary Table 3 Daily intake of energy and macronutrients (mean and standard deviation) at home among 3-5-year-old Finnish children, FoodStep Study

	Baseline		Follow-Up I		Follow-Up II		p value for interaction [a,b]
	**Intervention** n = 29	**Control** n = 47	**Intervention** n = 33	**Control** n = 39	**Intervention** n = 31	**Control** n = 37	
	**Mean (SD)**	**Mean (SD)**	**Mean (SD)**	**Mean (SD)**	**Mean (SD)**	**Mean (SD)**	
Energy (MJ)	5.61 (1.33)	5.87 (1.37)	5.33 (1.53)	5.16 (1.28)	4.97 (1.48)	5.41 (1.59)	ns
Protein (g/MJ)	9.21 (0.91)	9.07 (1.18)	9.21 (1.23)	9.07 (1.28)	9.24 (1.04)	8.88 (1.02)	ns
Protein (%E)	15.7 (1.55)	15.4 (2.00)	15.7 (2.08)	15.4 (2.18)	15.7 (1.77)	15.1 (1.74)	ns
Fat (g/MJ)	8.39 (0.98)	7.93 (1.00)	8.21 (0.93)	7.86 (0.91)	7.83 (1.03)	7.87 (0.89)	ns
Fat (%E)	31.0 (3.62)	29.4 (3.71)	30.4 (3.43)	29.1 (3.37)	29.0 (3.82)	29.1 (3.31)	ns
SFA (%E)	12.2 (2.34)	11.7 (2.09)	11.8 (1.80)	11.6 (1.86)	11.2 (2.22)	11.6 (1.98)	ns
MUFA (%E)	10.6 (1.34)	9.97 (1.64)	10.5 (1.61)	9.88 (1.52)	9.91 (1.56)	9.83 (1.45)	ns
PUFA (%E)	4.87 (0.95)	4.49 (1.01)	4.75 (0.94)	4.38 (0.97)	4.70 (0.83)	4.40 (0.98)	ns
n-3 (%E)	1.12 (0.26)	0.99 (0.23)	1.09 (0.27)	0.96 (0.24)	1.08 (0.21)	0.98 (0.22)	ns
n-6 (%E)	3.63 (0.74)	3.39 (0.81)	3.55 (0.73)	3.31 (0.77)	3.51 (0.67)	3.31 (0.80)	ns
Cholesterol (g/MJ)	26.4 (5.46)	25.7 (7.08)	26.7 (6.21)	25.7 (7.56)	27.6 (10.6)	26.2 (6.83)	ns
Carbohydrates (g/MJ)	29.3 (1.99)	30.2 (2.49)	29.7 (2.06)	30.5 (2.26)	30.4 (2.34)	30.6 (2.34)	ns
Carbohydrates (%E)	51.1 (3.47)	52.9 (4.30)	51.8 (3.46)	53.2 (3.81)	53.1 (4.02)	53.5 (3.92)	ns
Sucrose (g/MJ)	6.80 (1.57)	6.85 (1.97)	7.13 (1.68)	6.92 (2.06)	7.50 (1.85)	7.34 (1.86)	ns
Sucrose (%E)	11.6 (2.67)	11.7 (3.35)	12.1 (2.86)	11.8 (3.51)	12.8 (3.15)	12.5 (3.16)	ns
Dietary fibre (g/MJ)	3.64 (0.87)	3.80 (0.82)	3.44 (0.65)	3.61 (0.73)	3.55 (0.74)	3.70 (0.79)	ns
IDF (g/MJ)	2.40 (0.69)	2.53 (0.66)	2.26 (0.51)	2.37 (0.56)	2.35 (0.60)	2.46 (0.62)	ns
SDFP (g/MJ)	0.86 (0.18)	0.90 (0.18)	0.83 (0.14)	0.87 (0.17)	0.84 (0.15)	0.87 (0.15)	ns
SDFS (g/MJ)	0.34 (0.08)	0.34 (0.09)	0.33 (0.06)	0.34 (0.08)	0.33 (0.07)	0.35 (0.10)	ns

a) Bonferroni-corrected p values, multilevel linear mixed models were used with sex as fixed effects, and ID and region of the ECEC centre as random effects

b) Not significant (ns)

Supplementary Table 4 Daily intake of selected nutrients (mean and standard deviation) at home among 3-5-year-old Finnish children, FoodStep Study

	**Baseline**		**Follow-Up I**		**Follow-Up II**		**p value for interaction[a,b]**
	**Intervention**<br><br>**n = 29**	**Control**<br><br>**n = 47**	**Intervention**<br><br>**n = 33**	**Control**<br><br>**n = 39**	**Intervention**<br><br>**n = 31**	**Control**<br><br>**n = 37**	
	**Mean (SD)**	**Mean (SD)**	**Mean (SD)**	**Mean (SD)**	**Mean (SD)**	**Mean (SD)**	
Vitamin A (µg RAE/MJ)	124 (37.2)	128 (43.9)	129 (46.1)	127 (43.3)	130 (48.1)	120 (42.7)	ns
Vitamin C (mg/MJ)	19.0 (8.14)	19.5 (7.21)	19.9 (6.69)	18.0 (5.68)	19.7 (6.99)	17.9 (7.07)	ns
Vitamin D (µg/MJ)	3.36 (1.10)	3.48 (1.23)	3.73 (1.24)	3.68 (1.54)	3.82 (1.24)	3.28 (1.21)	ns
Vitamin E (mg/MJ)	1.41 (0.42)	1.42 (0.40)	1.55 (0.48)	1.42 (0.48)	1.48 (0.44)	1.33 (0.41)	ns
Thiamin (mg/MJ)	0.19 (0.06)	0.21 (0.07)	0.22 (0.08)	0.21 (0.08)	0.21 (0.07)	0.19 (0.07)	ns
Riboflavin (mg/MJ)	0.32 (0.09)	0.34 (0.10)	0.36 (0.12)	0.34 (0.11)	0.35 (0.10)	0.31 (0.09)	ns
Niacin (mg/MJ)	3.92 (0.71)	4.01 (0.93)	4.27 (1.05)	4.05 (1.02)	4.16 (0.92)	3.80 (0.89)	ns
Vitamin B6 (mg/MJ)	0.30 (0.10)	0.33 (0.10)	0.35 (0.11)	0.33 (0.10)	0.33 (0.11)	0.31 (0.09)	ns
Vitamin B12 (mg/MJ)	0.74 (0.18)	0.70 (0.19)	0.79 (0.23)	0.70 (0.20)	0.76 (0.21)	0.65 (0.17)	ns
Folate (µg/MJ)	37.6 (12.3)	38.5 (12.4)	41.5 (14.4)	37.7 (13.8)	40.6 (13.3)	34.8 (11.9)	ns
Potassium (mg/MJ)	440 (61.3)	459 (77.0)	452 (66.1)	454 (69.7)	451 (77.0)	441 (61.9)	ns
Calcium (mg/MJ)	180 (49.2)	182 (46.8)	189 (61.0)	182 (52.5)	185 (57.9)	169 (44.1)	ns
Magnesium (mg/MJ)	43.9 (5.58)	45.9 (5.49)	44.4 (5.22)	44.9 (5.14)	44.6 (5.58)	43.1 (4.20)	ns
Salt (g/MJ)	0.79 (0.09)	0.78 (0.10)	0.77 (0.11)	0.79 (0.09)	0.79 (0.10)	0.78 (0.07)	ns
Iron (mg/MJ)	1.65 (0.46)	1.84 (0.71)	1.80 (0.55)	1.81 (0.66)	1.76 (0.53)	1.65 (0.47)	ns
Selenium (µg/MJ)	9.77 (1.83)	9.87 (2.45)	10.6 (2.53)	10.0 (2.66)	10.4 (2.28)	9.31 (2.18)	ns
Phosphorus (mg/MJ)	189 (28.2)	193 (30.2)	192 (34.4)	191 (34.5)	193 (33.4)	183 (26.4)	ns
Iodine (µg/MJ)	31.5 (5.67)	33.3 (7.41)	33.7 (6.74)	33.5 (8.30)	33.7 (6.41)	30.9 (5.89)	ns
Zinc (mg/MJ)	1.59 (0.40)	1.70 (0.50)	1.77 (0.53)	1.71 (0.53)	1.70 (0.49)	1.55 (0.47)	ns

[a)] Bonferroni-corrected p values, multilevel linear mixed models were used with sex as fixed effects, and ID and region of the ECEC centre as random effects

[b)] Not significant (ns)
